# Editorial: The Covid-19 and TB syndemic: differences and similarities

**DOI:** 10.3389/fimmu.2023.1340231

**Published:** 2023-11-29

**Authors:** Stefan H. E. Kaufmann, Alex Sigal, Birgit Sawitzki, Alan Sher

**Affiliations:** ^1^ Director Emeritus, Max Planck Institute for Infection Biology, Berlin, Germany; ^2^ Emeritus Group of Systems Immunology, Max Planck Institute for Multidisciplinary Sciences, Göttingen, Germany; ^3^ Hagler Institute for Advanced Study, Texas A&M University, College Station, TX, United States; ^4^ Africa Health Research Institute, Durban, South Africa; ^5^ Centre for the AIDS Programme of Research in South Africa, Durban, South Africa; ^6^ School of Laboratory Medicine and Medical Sciences, University of KwaZulu-Natal, Durban, South Africa; ^7^ Translational Immunology, Berlin Institute of Health & Charité University Medicine, Berlin, Germany; ^8^ Research Center ImmunoSciences, Laboratory of Parasitic Diseases, National Institute of Allergy and Infectious Diseases, Bethesda, MD, United States

**Keywords:** tuberculosis, immunity, COVID-19, vaccination, BCG

Three years ago, in 2020, when the Covid-19 outbreak in China became a pandemic, the World Health Organization (WHO) declared Covid-19 a global health emergency (1). The consequences of this declaration were profound and led to the worldwide mobilization of virtually unlimited funding for needed intervention strategies. As a result, in 2023, the WHO could announce the end of Covid-19 as a global health emergency. Thirty years ago, when tuberculosis (TB) caused 3 million deaths per year, an earlier declaration by WHO came to a similar conclusion, namely that TB was a global health emergency (2). This announcement was more or less ignored and the WHO could never declare the end of TB as a global health emergency. Probably one major reason was that Covid-19 was a pandemic equally affecting countries worldwide while TB is primarily a disease of low-to-middle-income countries.

Consequently, financial support for TB has remained insufficient with support for research and development (R&D) in the order of 1 billion USD per year in the early 2020s. In comparison, the 3 years of the Covid-19 pandemic witnessed financial support for R&D in the order of 100 billion USD. There are other dissimilarities, but also similarities, between TB and Covid-19, and they are discussed in this Frontiers in Immunology Research Topic. The emphasis here is on the lessons learned from the successful handling of the Covid-19 pandemic for mitigating the dire TB crisis. A further issue addressed is whether interactions occur between the two infections, with one pathogen blocking or promoting the disease induced by the other.

In describing epidemiologic aspects of Covid-19 and TB, Falzon et al. emphasize the negative impact of Covid-19 on TB worldwide, which led to a reversal of the slight decline in TB morbidity and mortality before the Covid-19 crisis.

Booysen et al. provide an update on immune interactions between the two causative agents, SARS-CoV-2 and *Mycobacterium tuberculosis* (Mtb). They conclude that coinfections could impair immunity to SARS-CoV-2 due to elevated inflammation. Furthermore, they find a lack of evidence for a beneficial effect of the TB vaccine, BCG, against Covid-19. These issues have been further analyzed in experimental animal studies.

Hilligan et al. show in an experimental animal model that only intravenous administration of BCG can provide protection against Covid-19 and Baker et al. reveal in an experimental mouse model that prior infection with SARS-CoV-2 did not affect subsequent Mtb infection, whereas prior Mtb infection restricted replication of SARS-CoV-2 after subsequent challenge.

Aiello et al. further elaborate on the immune response against Mtb and SARS-CoV-2 in humans with an emphasis on the initial stage.

Allué-Guardia et al. elaborate on another similarity between TB and Covid-19, namely the increased susceptibility and the heightened burden of disease in the elderly. As they point out, a better understanding of mechanisms underlying TB, Covid-19, and other respiratory infections should be harnessed for the design of future intervention strategies to increase the health span of the elderly.

Shaw et al. discuss one possible mechanism that could operate as a disease magnifier in infectious diseases, such as Covid-19 and TB, namely myeloid-deprived suppressor cells.

Kaufmann provides an overview of the current status of vaccine R&D against TB and Covid-19 and provides possible explanations for the differential speed of R&D for Covid-19 vs. TB vaccine development with an emphasis on the mechanisms underlying immune protection that were mobilized for the development of preventive measures against the two diseases.

Corleis et al. provide an overview of different animal models harnessed for TB and Covid-19 investigation and underline an optimization of models, notably for pulmonary infectious diseases such as Covid-19 and TB. A complementary, rather than alternate approach, would be controlled human challenge studies.

Morrison et al. describe the state-of-the-art and potential future developments for controlled human infection models for SARS-CoV-2 and TB. Such models could also provide models for newly emerging pathogens.

The rapid and highly efficient response against Covid-19 has demonstrated the importance of public awareness of the threat of infectious diseases including not only newly emerging but also current threats. Hopefully, the lessons learned from the response to Covid-19 will also impact efforts toward better control of TB. In September 2023, the United Nations convened a high-level meeting on TB, which resulted in a commitment to provide life-saving treatment for up to 45 million people between 2023 and 2027, including up to 4.5 million children and up to 1.5 million people with drug-resistant TB (3, 4).

Furthermore, they endorsed preventive treatment for up to 45 million people between 2023 and 2027, including 30 million household contacts of TB patients and children, and 15 million people living with HIV. To achieve these goals, an increase in annual global TB funding to 22 billion USD annually by 2027 and to 35 billion USD by 2030 was promised. This also included the mobilization of 5 billion USD per year by 2027 for R&D for TB.

Even though the UN declaration did not include clear accountability of the signatories, it would be important for the whole world to accomplish these goals since, without such measures, an estimated 24 million deaths will be caused by TB by 2050, leading to economic losses on the order of 13 trillion USD. The example of Covid-19 has shown that this level of financial support can make a difference (5). Therefore, while the effects of the Covid-19 pandemic on TB control have been predominantly negative, we learned that high investment, both in labor and funding resources, brings impactful results. We hope this lesson will be transferred from a new pandemic to an old one.

## Author contributions

SK: Writing – original draft, Writing – review & editing. ASi: Writing – review & editing. BS: Writing – review & editing. ASh: Writing – review & editing.
